# Environmental drivers of the allergenic load caused by *Ambrosia artemisiifolia* pollen and its major allergen Amb a 1 in the atmosphere

**DOI:** 10.1007/s00484-025-02932-5

**Published:** 2025-04-29

**Authors:** Jana Ščevková, Matúš Žilka, Jozef Dušička, Zuzana Vašková, Jozef Kováč, Eva Zahradníková

**Affiliations:** 1 Faculty of Natural Sciences, Department of Botany, Comenius University, Révová 39, Bratislava, 81102 Slovakia; 2 Faculty of Mathematics, Physics and Informatics, Department of Applied Mathematics and Statistics, Comenius University, Mlynská dolina, Bratislava, 84248 Slovakia

**Keywords:** Ragweed pollen, Amb a 1 allergen, Pollen allergen potency, ELISA, Meteorological parameters, Air pollutants

## Abstract

Understanding the environmental factors influencing the allergenicity of *Ambrosia artemisiifolia* pollen is crucial for effective allergy prevention. This study, conducted from 2019 to 2022 in Bratislava, Slovakia, utilised a Hirst-type Burkard pollen trap and a Burkard multi-vial cyclone sampler to measure airborne ragweed pollen (by microscopic analysis) and Amb a 1 allergen concentrations (by ELISA), enabling the calculation of pollen allergen potency (PAP). The data analysis showed that annual pollen and allergen levels are affected by meteorological conditions: sunny and dry weather in May accelerated pollen production, while dry conditions in August increased allergenic potency during the main flowering phase. Based on Spearman’s correlation analysis, daily allergen levels were significantly associated with pollen concentrations. Regression analysis confirmed the influence of environmental factors on pollen, allergen and PAP levels. Pollen concentration showed a positive association with temperature and wind speed but a negative association with precipitation. Relative humidity was the only meteorological factor negatively linked to allergen levels, while temperature had a negative impact on PAP. The most relevant air pollutants were CO and SO_2_, which increased allergen levels and PAP, respectively.

**Supplementary Information:**

The online version contains supplementary material available at 10.1007/s00484-025-02932-5.

## Introduction

The genus *Ambrosia* comprises 46 species worldwide, of which only *Ambrosia artemisiifolia* L. occurs in Slovakia (Govaerts 2024). Native to North America, *A. artemisiifolia* L. (common ragweed, hereafter referred to as ragweed) is considered an invasive plant species in most European countries, including Slovakia (Schaffner et al. 2020; Hrabovský et al. 2024). In addition to the adverse effects of ragweed on biodiversity and crop yields, its pollen grains cause allergic respiratory diseases (pollinosis) in sensitive individuals, with typical symptoms including rhinoconjunctivitis and asthma (Ihler and Canis 2015). Almost 16 million people in Europe are estimated to be sensitised to ragweed pollen (Schaffner et al. 2020), with late summer and autumn being considered the main pollination period (Sikoparija et al. 2017).

Ragweed is an anemophilous species, generating up to 3 billion small (10–25 μm) pollen grains per plant (Fumanal et al. 2007; Sam et al. 2020). It is widespread in southern and central Slovakia, where the daily airborne pollen concentration can reach up to 500 pollen/m^3^ (Ščevková et al. 2024), a level 25 times higher than the threshold value that triggers initial symptoms in sensitised individuals (Stępalska et al. 2008). In Bratislava, the area with the highest ragweed infestation in Slovakia, the mean value of the Seasonal Pollen Integral (SPIn) is 1,779 pollen*day/m^3^ (Ščevková et al. 2024).

Ragweed pollen contains several allergenic molecules (Allergen nomenclature; http://www.allergen.org/), of which Amb a 1 has been identified as one of the major allergens (Adolphson et al. 1978; Wopfner et al. 2005; Oberhuber et al. 2008). Amb a 1, an acidic, non-glycosylated 38 kDa protein from the pectate lyase family, is recognised as the key allergen responsible for over 95% of allergic responses in sensitised individuals (King et al. 1964; Gadermaier et al. 2008). The Amb a 1 protein is predominantly bound to the pollen intine layer (Knox and Heslop-Harrison 1971). It is possible to determine the Pollen Allergen Potency (PAP) based on its quantity in the pollen grain. The PAP value is not constant, but changes within and between seasons and locations based on environmental conditions; the exact mechanism of this variability is still unknown (Tegart et al. 2021). From this perspective, aeropalynological monitoring, which considers the concentration of pollen grains in the air as a proxy for aeroallergen exposure, is not always sufficient and needs to be supplemented with the quantification of allergenic proteins in the air (Buters et al. 2012, 2015; Galan et al. 2013; Plaza et al. 2016).

The number of people suffering from some form of seasonal respiratory allergy is increasing yearly, with this growing trend being more pronounced in densely populated urban areas (Sedghy et al. 2018). The precise causes of this condition remain unclear; however, the trend is frequently linked to elevated levels of air pollution from anthropogenic sources and environmental factors specific to urban areas (de Lira-Quezada et al. 2024). These include the distinct microclimatic conditions amplified by the urban heat island phenomenon (Rizwan et al. 2008), gradually intensified by the recent climate changes (Kamal et al. 2023). These changes, particularly atmospheric warming and increasing drought, are also more pronounced in urban areas. Elevated temperatures can to a certain extent stimulate growth in some plants, leading to higher pollen grain production (Ziska et al. 2003), but together with drought may also act as a stressor on certain pollen-producing plant species. This can enhance the allergenicity of their pollen grains by increasing the expression or inducing the secretion of new allergenic proteins (Buters et al. 2008) or by modulating allergen-IgE binding activity (Gentili et al. 2019). The same effect was observed for high concentrations of air pollutants such as nitrogen dioxide (NO_2_), carbon monoxide (CO), ozone (O_3_), sulphur dioxide (SO_2_) and particulate matter in urban areas, associated mostly with traffic (Ghiani et al. 2012; Sousa et al. 2012; Zhao et al. 2016; Hinge et al. 2017; Sedghy et al. 2018).

Air pollutants can also alter the morphological structure of the pollen’s outer layer thus promoting the release of allergenic molecules (Ouyang et al. 2016). Additionally, pollutants attached to intact pollen, allergen-carrying pollen fragments, or free allergens can form complex particles that may amplify their adverse effects on human health by stimulating IgE synthesis and exacerbating allergy symptoms (Brito et al. 2007; D’Amato et al. 2010).

This study aimed to reveal the relationship between the airborne concentrations of *Ambrosia* pollen and Amb a 1 allergen and to identify the environmental drivers of allergen exposure, aiding in the prediction and management of allergic diseases caused by ragweed pollen.

## Materials and methods

### Study area

The study was conducted in the capital of Slovakia, Bratislava, located in the southwest of the country in Central Europe. The city has a population of 477 thousand (Statistical yearbook of the capital of the SR Bratislava, 2023) and covers an area of 367.6 km^2^. It is situated at the base of the Malé Karpaty Mts., between the Záhorská nížina and Podunajská nížina lowlands with elevations ranging from 126 to 514 m above sea level. The climate is classified as Cfb of the Köppen–Geiger climate types (warm temperate, fully humid with warm summers (Kottek et al. 2006). For the 1983‒2023 period, the annual mean temperature was 10.9 °C and the average annual precipitation total was 703.4 mm (Slovak Hydrometeorology Institute, Meteorological observatory Bratislava ‒ Mlynská dolina). Of the four years analysed, 2019 was the warmest (12.2 °C), 2020 the rainiest (1,141 mm), 2021 the coldest (10.8 °C), and 2022 the driest (830.4 mm).

In Bratislava, *Ambrosia artemisiifolia* primarily occurs in unmaintained and disturbed areas, such as construction sites, roadsides, agricultural land, and industrial zones. The Podunajská nížina Lowland, which extends into the city, provides ideal conditions for the spread of ragweed due to its climate, especially along road and rail corridors. Recent climate changes also enable its spread northward into cooler regions of Slovakia (Hrabovský et al. 2024).

### Aerobiological analysis

The airborne concentrations of ragweed pollen and Amb a 1 allergen were measured using a Hirst-type Burkard pollen trap and a Burkard multi-vial cyclone sampler, respectively. Sampling was conducted daily from June 1 to October 31 over four consecutive years (2019–2022), totalling 153 sampling days per year. The equipment was placed on the roof of the Comenius University Science Park building (48.149444° N, 17.073333° E), 18 m above ground level.

The Hirst-type sampler operated with a constant air intake rate of 10 l/min and a drum rotation speed of 2 mm/h (Hirst 1952). The pollen grains drawn through a narrow slit (14 × 2 mm) were captured on Melinex tape fixed on a rotating drum coated with Vaseline wax. After collection, the tape was sectioned into seven segments representing daily samples and placed on microscope slides. To determine the average daily pollen concentration in the air (pollen/m³), we analysed the slides using the twelve vertical transect method at 400× magnification. This evaluation followed the detailed methodology outlined by Lacey and West (2006). Ragweed pollen grains were identified using their unique morphological features (Grant Smith 2000) and reference collections of pollen grains.

The multi-vial cyclone sampler drew airborne bioparticles through a narrow orifice into the small cyclone and collected them as dry samples in seven 1.5 ml Eppendorf vials, each vial representing a 24-hour exposure period. Ragweed Amb a 1 protein was extracted from the samples using 120 µl of a buffer solution composed of 50 mM phosphate buffer (pH 7.4), 150 mM NaCl, 125 mM ammonium bicarbonate, 3 mM EDTA, and 0.005% Tween 20. The extraction process was performed at room temperature with continuous stirring for 2 h. Following extraction, the samples were centrifuged at 2,000 g for 10 min to pellet the material and then stored at -20 °C. Amb a 1 proteins were quantified through an enzyme-linked immunosorbent assay (ELISA) using the Amb a 1 2.0 EP kit (Indoor Biotechnologies) according to the manufacturer’s instructions. The final absorbance values were measured at 450 nm using a HiPo Microplate Photometer MPP-96 (Biosan). The daily average concentrations of Amb a 1 were reported in pg/m³.

The ragweed main ragweed pollen season (MPS) for each study year was determined using the criteria set by Nilsson and Persson (1981). According to this method, the MPS begins when the accumulated sum of ragweed mean daily pollen concentrations reaches 5% of the APIn (annual pollen integral) and ends when 95% of the APIn is recorded. For our analyses, we extended this interval by ten days before and ten days after to account for the long-distance transport of pollen grains and allergen. This interval is referred to as “extended pollen season”, the interval of ten days before the start of MPS as “pre-season”, the span of MPS as “in-season”, and ten days after the final day of MPS as “post-season”.

The intensity of the MPS was assessed based on the following characteristics: (1) seasonal pollen integral (SPIn, the sum of the mean daily pollen concentrations during the MPS), (2) peak pollen value, (3) high pollen days (HPD, number of days when the pollen concentration reached a daily average greater than 20 pollen/m^3^, the threshold at which initial allergic symptoms manifest in sensitive individuals (Stępalska et al. 2008), (4) seasonal allergen integral (SAIn, the sum of the mean daily allergen concentrations during the MPS), (5) peak allergen value, (6) high allergen days (HAD, number of days when the allergen concentration reached a daily average greater than 50 pg/m^3^– the recorded average allergen concentration corresponding to 20 pollen/m^3^) and (7) pollen allergen potency (PAP, the atmospheric ratio of the daily allergen and pollen concentrations, given in pg Amb a 1/pollen).

### Environmental parameters

The study considered meteorological factors (air temperature, sunshine, rainfall, relative air humidity, and wind speed) along with air pollutants (CO– carbon monoxide, NO_2_– nitrogen dioxide, SO_2_– sulfur dioxide, O_3_– ozone, BTX– mixtures of benzene, toluene, and the three xylene isomers), with their average values during the studied years shown in Table 1. Meteorological data were collected from the Bratislava - Mlynská dolina observatory, located 300 m from the pollen and allergen monitoring station. Daily air pollutant levels were averaged from four monitoring stations (Mamateyova, Trnavské mýto, Kamenné námestie, and Jeséniova) across Bratislava, based on data availability. These stations, located 2.5 to 4 km away from the aerobiological monitoring station by air, are part of the Slovak air quality monitoring network, operated by the Slovak Hydrometeorological Institute.

**Table 1:** The characteristics of *Ambrosia artemisiifolia* main pollen season in Bratislava (2019 − 2022)

Characteristics	2019	2020	2021	2022
***Ambrosia*** **pollen data**				
Pollen season start	14 Aug	16 Aug	22 Aug	15 Aug
Pollen season end	22 Sep	21 Sep	3 Octo	25 Sep
Season length (days)	40	37	43	42
Seasonal Pollen Integral (pollen*day/m^3^)	1,735	2,072	1,904	1,950
Peak pollen value (pollen/m^3^)	203	315	375	256
Peak pollen day	28 Aug	28 Aug	9 Sep	8 Sep
High pollen days (number)^a^	24	23	19	19
**Amb a 1 allergen data**				
Seasonal Allergen Integral (pg*day/m^3^)	3,757.6	1,269.2	567.5	3,571.9
Peak allergen value (pg/m^3^)	495.2	248.6	130.0	891.3
Peak allergen day	31 Aug	30 Aug	9 Sep	1 Sep
High allergen days (number)^b^	24	6	3	13
Pollen Allergen Potency (pg Amb a 1/pollen)^c^	8.7	1.5	1.8	5.0
**Meteorological data** ^c^				
Temperature (°C)	19.2	19.5	16.7	18.3
Sunshine (h)	7.1	7.3	6	5.8
Relative humidity (%)	66.6	67.6	75.5	83.2
Precipitation (mm)	4.3	3.3	5.7	3.6
Wind speed (m/s)	1.33	1.35	1.27	1.28
**Air pollution data**^c^**(**in µg/m^3^)				
O_3_	69.5	63.1	60.6	65
CO	353.1	280.1	279.9	190.7
NO_2_	20.1	20.2	18	14.1
SO_2_	8.3	2.1	5.2	2.8
BTX	1.1	0.4	0.5	0.2

^a^pollen concentration ˃ 20 pollen/m^3^

^b^allergen concentration ˃ 50 pg/m^3^

^c^daily average value

### Data analysis

To determine the influence of environmental factors on the SPIn value, we focused on the weather conditions and air pollutants in May, when the effect on *Ambrosia* biomass growth in Central Europe and subsequent pollen production is highest (Knolmajer et al. 2024). Regarding the SAIn and PAP values, we additionally considered the environmental conditions in August, during the optimum of ragweed flowering in Slovakia (Ščevková et al. 2024), when allergenic molecules are produced in pollen grains during the final stage of maturation in the anthers (Turcich et al. 1993). We compared the values of all these parameters with the calculated four-year average. To ensure comparability among these averages, we standardised each variable before analysis. This process involved subtracting the four-year average from each value within a variable and then dividing by the four-year standard deviation, placing the averages on a consistent scale.

The strength of the association between pollen and allergen concentrations during the extended pollen season was assessed using a nonparametric Spearman correlation test. Linear regression was used to examine the relationships between meteorological variables and (i) pollen concentration, (ii) allergen concentration, and (iii) PAP. To account for interannual variability, a categorical variable “year” was included in the model, with levels representing the years 2019, 2020, 2021 and 2022. We also considered an indicator variable “consecutive precipitation” (consPrecip.) expressing whether there were several rainy days in a row (if there were at least two rainy days in a row, the value of the variable was 1, otherwise it was 0). In addition, we considered air pollutants and pollen concentration as explanatory variables for allergen concentration, while air pollutants were also considered an explanatory variable for PAP value. Finally, for each numerical explanatory variable, we also considered time lags ranging from 0 to 6 days. The choice of time lag was made individually for each variable to maximise Spearman’s correlation with the response variable.

First, we started with an ordinary regression i.e. with the model of the form

$$\:\text{y}=\text{X}{\upbeta\:}+{\upepsilon\:,}$$

where $\:y$ represents a vector of observed values (response variable), $\:X$ is a design matrix of explanatory variables, $\:{\upbeta\:}$ is an estimated vector of regression coefficients and $\:{\upepsilon\:}$ represents a vector of error terms, which we assume to have a normal distribution with uncorrelated components with zero mean value and constant variance (i.e. $\:{\upepsilon\:}\sim\:N(0,{{\upsigma\:}}^{2}I)$). However, the assumption of uncorrelatedness is often naive in time series, as can be seen from the analysis of the residuals of such models in Figs. S1-3– in the case of uncorrelated errors, all vertical lines in the left plot (Autocorrelation function of residuals) should be close to zero (in the blue band or at least close enough to it) and all the *p*-values of the Ljung-Box test in the bottom plot should be above 0.05. In addition, we can see a strong deviation from normality in the right plot of these figures (Normal Q-Q plot).

**Fig. 1:**
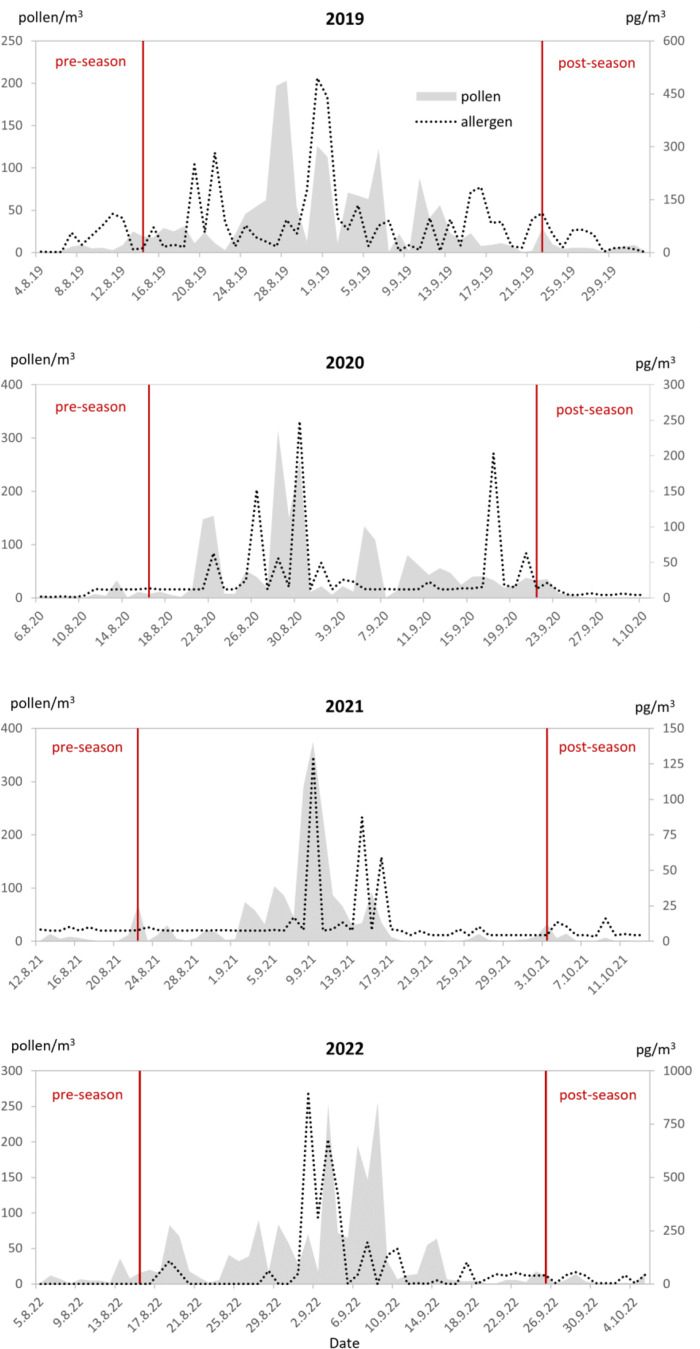
The airborne levels of *Ambrosia* pollen and Amb a 1 allergen in Bratislava, 2019‒2022. The start and end of the MPS are marked by red lines

**Fig. 2:**
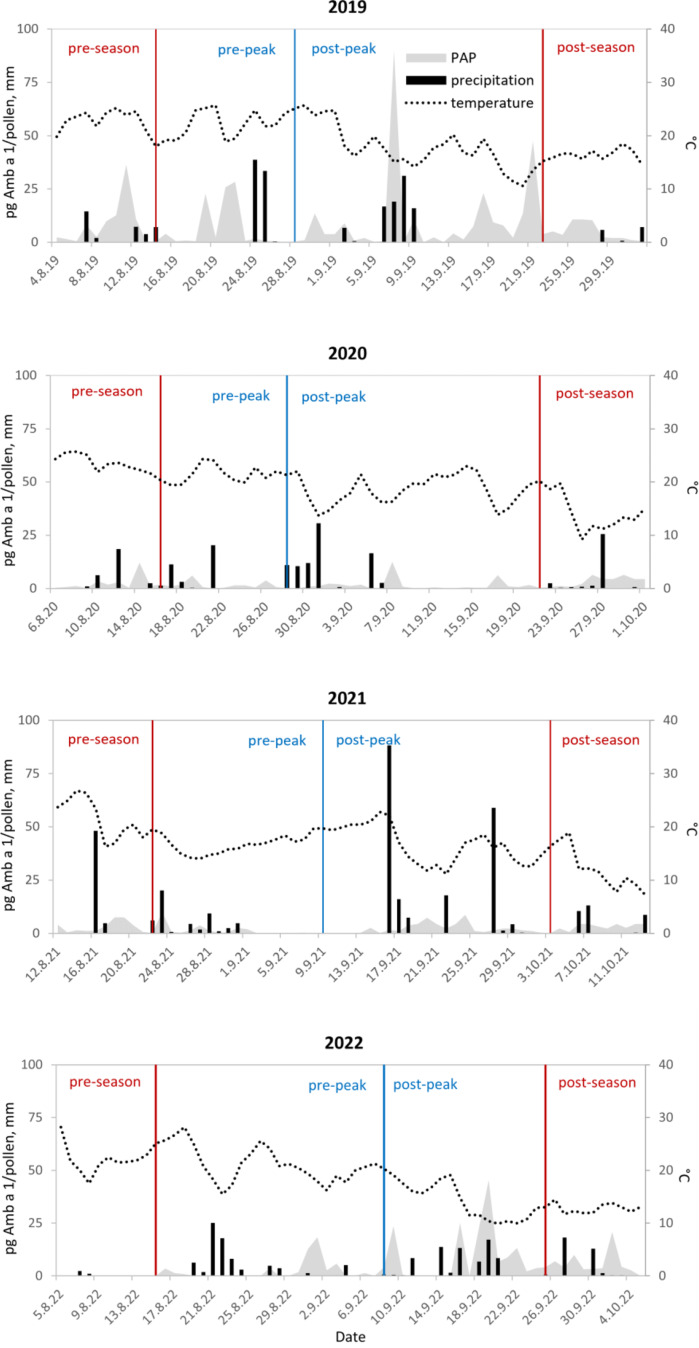
Daily variation in precipitation totals, mean air temperature and ragweed pollen allergen potency (PAP) levels in Bratislava, 2019‒2022. The start and end of the MPS are marked by red lines and the peak pollen date of the MPS by a blue line

**Fig. 3:**
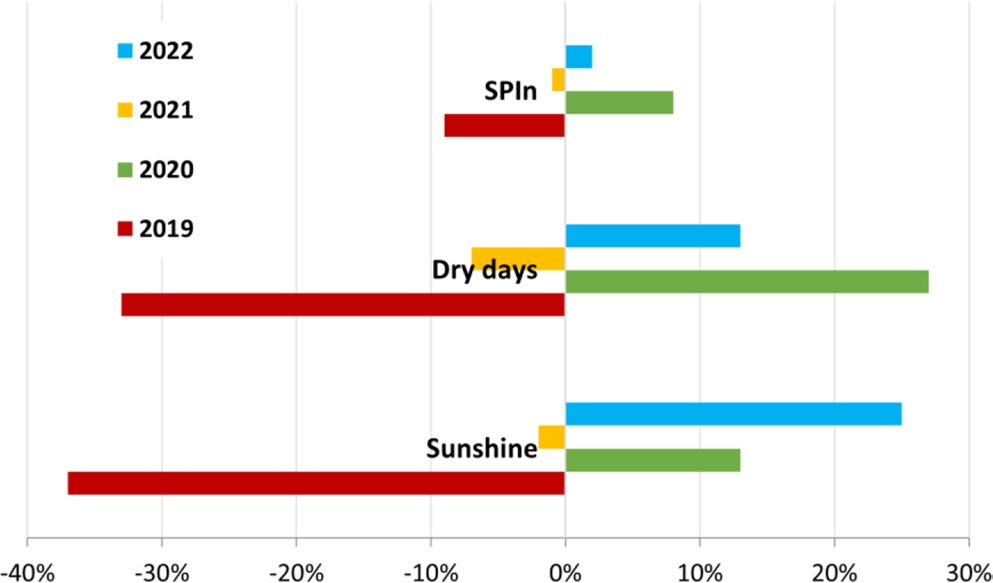
Increase/decrease in the levels of SPIn and significant environmental parameters (number of dry days and sunshine) in Bratislava in May over the individual years compared to the four-year average values (2019 − 2022)

To address these issues, two adjustments were made to the model. First, we applied a logarithmic transformation to the response variable (or a $\:{log}\left(y+1\right)$ transformation in case the explanatory variable contained zeros), which improved the normality of the residuals. Second, we included an autoregressive moving average (ARMA) structure for the error term $\:{\upepsilon\:}$, choosing the ARMA order that minimised the number of parameters while eliminating autocorrelation in the residuals. This means that in the above model we have assumed $\:{\upepsilon\:}\sim\:N\left(0,{{\upsigma\:}}^{2}W\right)$, where $\:W$ is an autocorrelation matrix of some ARMA model dependent on a small number of parameters that we estimate along with the vector of regression coefficients $\:{\upbeta\:}$. Further details of this method can be found in Sect. 5.3 of Pinheiro and Bates (2000).

Analyses were performed using R software. The unknown parameters were estimated by the maximum likelihood method using the *gls* function from the *nlme* package. Variable selection was then performed based on the Akaike information criterion (AIC) by iteratively removing the least significant variables using the *stepAIC* function from the *MASS* package. Finally, in the final models, we checked the model assumptions on the normalised residuals (estimates of the values of $\:{W}^{-\frac{1}{2}}{\upepsilon\:}$, which should be close to $\:N\left(0,{{\upsigma\:}}^{2}I\right)$ distribution in our model). This was, as in the case of ordinary regression, done using the *sarima* function from the *astsa* package, where we simply checked whether the normalised residuals could be white noise. In Figs. S4-6, one can see the significant improvement in meeting the assumptions of the model.

**Fig. 4:**
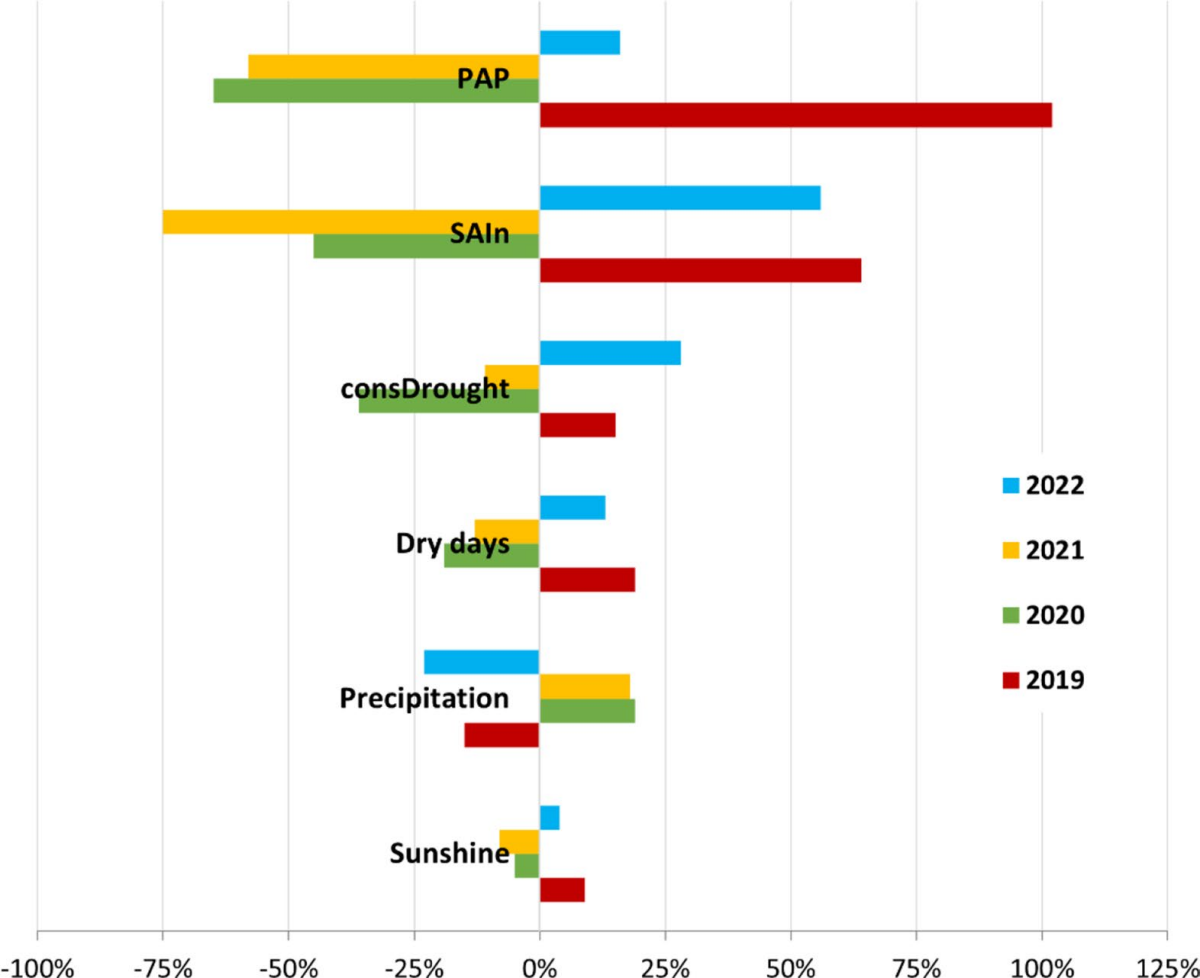
Increase/decrease in the levels of SAIn, pollen allergen potency (PAP) levels and significant environmental parameters (sunshine, precipitation, number of dry days and consDrought − number of consecutive days without precipitation) in Bratislava in August over the individual years compared to the four-year average values (2019 − 2022)

**Fig. 5:**
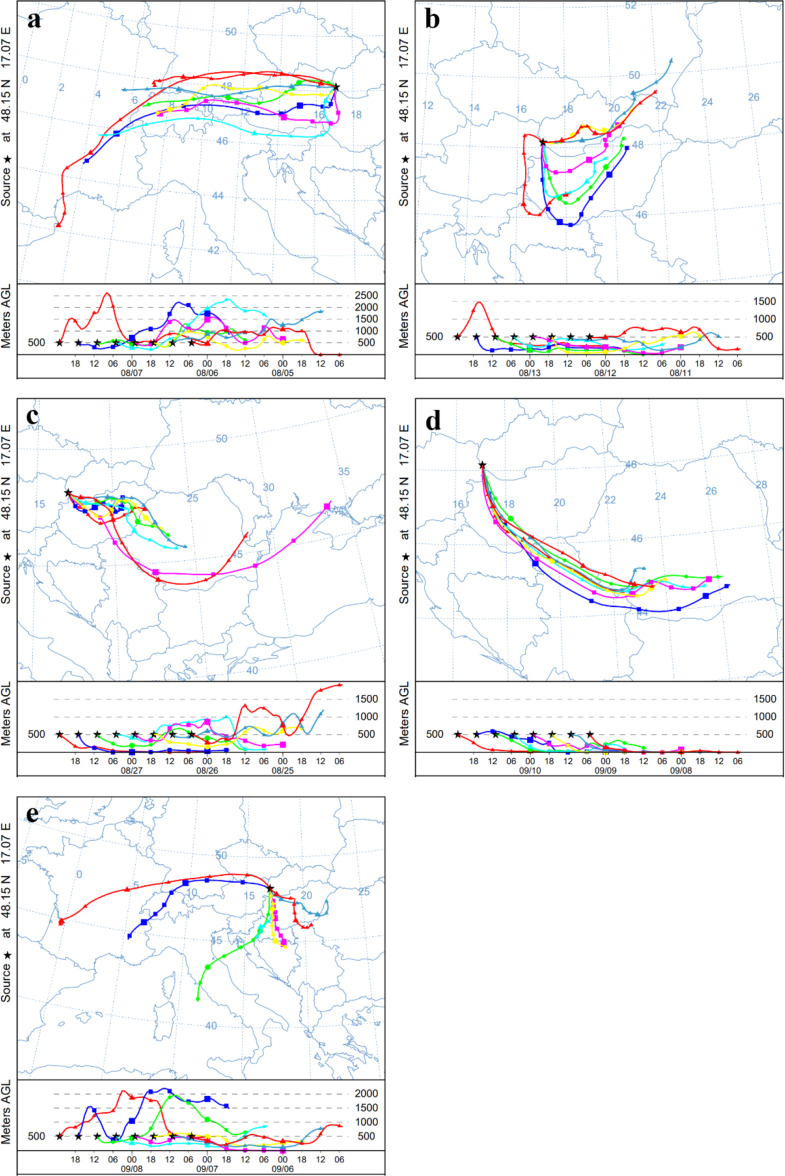
Maps illustrating 48-hour HYSPLIT back trajectories for selected high ragweed pollen or Amb a 1 allergen episodes, with air parcel endpoints at an altitude of 500 m are presented for the following LDT episodes: (**a**) 6th-7th August 2019, (**b**) 12th-13th August 2020, (**c**) 26th-27th August 2019, (**d**) 9th-10th September 2021, (**e**) 7th-8th September 2022

As the standard R² metric may not be appropriate for models with autocorrelation in the residuals, we report the Cox-Snell pseudo-R² instead. In this model, AIC takes preference before the *p*-value, i.e. *p*-values above 0.05 are acceptable if removing them would make the AIC worse.

### Back-trajectory analysis

To determine the origin of *Ambrosia* pollen and Amb a 1 allergen during the days with discrepancies in their concentrations, we used the Hybrid Single-Particle Lagrangian Integrated Trajectory (HYSPLIT) model to compute 48-hour back trajectories in three dimensions (latitude, longitude, and altitude). For each episode, eight trajectories were generated at six-hour intervals, based on meteorological data from the Global Data Analysis System (GDAS). Our approach was adapted from previous studies (Fernández-González et al. 2019; Grewling et al. 2022; Plaza et al. 2016; Rolph et al. 2017), with modifications in trajectory duration and altitude selection. Isentropic back trajectories of air masses were modelled for pollen and allergen peak episodes, arriving in Bratislava at 23:00 UTC. All trajectories were calculated from an altitude of 500 m above ground level.

## Results

The characteristics of the *Ambrosia* MPS and Amb a 1 allergen occurrence during the studied years are summarised in Table 1. The SPIn value was comparable over the four analysed years, ranging from 1,735 (2019) to 2,072 (2020) pollen*day/m³, similarly to the peak pollen values (from 203 pollen/m³ in 2019 to 375 pollen/m³ in 2021) and HPD values. In contrast, we observed significant seasonal variability in SAIn values: the Amb a 1 SAIn value in 2019 was five times higher than in 2021 (3,757.6 vs. 567.5 pg*day/m³). The peak allergen values and the number of HAD values had a similar broad range of variability (peak allergen value 891.3 pg/m³ in 2022 vs. 130 pg/m³ in 2021 and 3 HAD in 2021 vs. 24 in 2019) (Table 1; Fig. 1).

The ragweed MPS in the studied years started in mid-August and ended in the second half of September, except for 2021, when both dates were delayed by more than a week. Pollen and allergen levels peaked almost simultaneously in all years, except for 2022, when they were one week apart (Table 1; Fig. 1).

We also observed significant annual variability in the PAP value, from 1.5 pg Amb a 1/pollen in 2020 to 8.7 pg Amb a 1/pollen in 2019 (Table 1). In all four analysed years, the maximum daily PAP value occurred during the MPS, pre-peak in 2021 and post-peak in the other years. In 2019 and 2022, higher PAP values (˃ 20 pg Amb a 1/pollen) were also recorded during the pre- and post-season periods, respectively (Fig. 2).

To determine which environmental factors influence the SPIn value, we analysed the weather conditions and air pollutants in May. The most significant impact on SPIn was linked to drought and sunshine. We observed that the higher number of dry days and sunshine levels in May led to increased SPIn values, as in 2020 and 2022 (Fig. 3).

The conditions in August are a key factor in determining the SAIn and PAP values of *Ambrosia* pollen. The precipitation total, the number of dry days, consDrought (number of consecutive days without precipitation) and sunshine were the most significant factors influencing both values (Fig. 4).

The Spearman’s correlation analysis conducted over the four examined years showed a significant positive association between daily ragweed pollen and Amb a 1 levels (*r* = 0.366, *p* < 0.001).

Multiple regression analysis was used to determine how individual environmental factors impact the allergenic attributes of ragweed: daily airborne pollen and allergen concentrations, and PAP levels during the extended pollen season (Table 2). Regarding pollen concentration, a significant positive association was observed with temperature and wind speed and a negative association with consPrecip. Relative humidity was the only meteorological parameter associated with allergen concentration (negatively) while temperature had a negative association with PAP. Air pollutants also had a positive impact on allergen levels (1-day lag of O_3_ and 5-day lag of CO) and PAP (5-day lag of SO_2_).

**Table 2:** Significant environmental variables in multiple regression models for *Ambrosia* pollen and amb a 1 allergen concentrations and PAP during the extended pollen season in Bratislava, 2019 − 2022

	Variables	β Coeff.	Std. error	*p*-value	*R* ^2^
Pollen	Intercept	-1.1853	0.6498	0.0694	0.1858
	Temperature	0.1489	0.0284	< 0.0001	
	Wind speed	0.4397	0.1339	0.0012	
	consPrecip.	-0.421	0.169	0.0134	
Allergen	Intercept	1.8857	0.936	0.0451	0.0698
	Relative humidity	-0.0197	0.0072	0.0069	
	O_3_ (lag 1)	0.016	0.0064	0.0136	
	CO (lag 5)	0.0036	0.0018	0.0534	
PAP	Intercept	2.5771	0.4305	< 0.0001	0.1492
	Year 2020	-0.2977	0.2758	0.2816	
	Year 2021	-0.6323	0.2131	0.0033	
	Year 2022	-0.3595	0.2569	0.163	
	Temperature	-0.0803	0.0152	< 0.0001	
	SO_2_ (lag 5)	0.0666	0.0342	0.0525	

*β* coefficient of the variable “Year” expresses the average difference between the values in the given year to 2019

Environmental factors accounted for 18.6% of the data variation for *Ambrosia* pollen, 7.0% for Amb a 1 allergen and 14.9% for PAP (Table 2).

The analysis of backward trajectories confirmed long-distance transport of *Ambrosia* pollen and Amb a 1 allergen during the periods of discrepancies in their concentrations (Fig. 5). Notable trajectories from ragweed-infested areas lead mostly through Hungary and Romania during the days with high pollen concentrations and through the Rhône-Alpes region in France during high allergen concentration pre-season.

## Discussion

Although SPIn values showed no significant variability over the four years analysed, we observed that a higher number of dry days and increased sunshine hours in May led to higher SPIn. This is when the intensive vegetative growth of *Ambrosia artemisiifolia*, a C3 photosynthetic species (Fumanal et al. 2008), begins after germination (Knolmajer et al. 2024). However, the dependence of plant growth on every ecological factor is a curve, with a decline in extreme conditions. When the drought gets too intense, it affects *Ambrosia* pollen production negatively, as shown by Sheoran and Saini (1996) and Fang et al. (2010).

In contrast to SPIn, we observed significant year-to-year fluctuations in ragweed SAIn, which also affected PAP levels. Ghiani et al. (2016) attributed seasonal variations in ragweed PAP levels mainly to climatic factors such as temperature, humidity, and light during the plant’s growth and flowering phases. While SPIn is correlated with SAIn and, therefore, also influenced by the conditions in May, our results additionally show a significant influence of conditions in August during the optimum of ragweed flowering season and pollen production in the study area. As drought intensifies in August, Amb a 1 production in pollen increases, leading to higher SAIn and PAP levels. This is consistent with El Kelish et al. (2014), who found that elevated drought stress and CO_2_ modified the ragweed pollen transcriptome, affecting PAP. Drought stress increases Amb a 1 level in pollen, suggesting that changes in Amb a 1 concentration might help ragweed plants adapt and enhance their reproductive success (El Kelish et al. 2014). Amb a 1 protein, part of the pectate lyase family, supports pollen tube growth by breaking down the pollen cell wall and aiding its penetration through transmitting tissue (Zhao et al. 2016).

Ragweed pollen grains are known for being involved in long-distance transport (Smith et al. 2008; de Weger et al. 2016; Stępalska et al. 2020; Menut et al. 2021). Pollen grains transported in this manner are exposed to harsh conditions in the atmosphere, including dynamic air circulation and the impact of chemical pollutants. This exposure leads to the release of allergen-containing cytoplasm into the environment as a result of pollen grain rupture or extrusion through apertures (Caronni et al. 2021). Allergens are lighter than intact pollen grains, which gives them a lower sedimentation velocity, allowing them to remain airborne longer and be carried over greater distances (Visez et al. 2015). These free allergens are impossible to distinguish from pollen-bound ones in the cyclone sample analysis, and it is also not possible to discern empty ragweed pollen grains from atmospheric samples using optical microscopy, therefore the PAP value does not express the amount of allergen per single pollen grain as defined by Galán et al. (2017), but rather their atmospheric ratio (Tegart et al. 2021).

The average PAP value over the MPS ranged from 1.5 pg Amb a 1/pollen (2020) to 8.7 pg Amb a 1/pollen (2019), which is consistent with the results of studies from Poznań (Poland) and Bursa (Turkey), where the average daily PAP value recorded was 4.3 and 2.6 pg Amb a 1/pollen, respectively (Grewling et al. 2016a; Celenk 2019). From an allergological perspective, significant PAP values were observed not only in-season but also during the pre- and post-season periods, with a maximum average daily value of 8.5 pg Amb a 1/pollen in the pre-season (2019) and 6.3 pg Amb a 1/pollen in the post-season (2022).

During the pre-season period, airflow from the south carries pollen grains and allergenic proteins into the air over Bratislava from Hungary, where the ragweed pollen season begins earlier (Magyar et al. 2022). Because of the longer stay of non-pollen-bound allergens in the atmosphere, certain weather conditions may bring more allergens than pollen grains into the studied area. The post-season period typically aligns with autumn, when Bratislava usually experiences more rainfall and stronger winds than in summer. Thus, higher PAP values during this time may be linked to (i) delayed pollen release from anthers, leading to increased synthesis of allergenic proteins within the pollen grains (Maya-Manzano et al. 2022); (ii) pollen rupture in the anther or directly in the air due to osmotic shock (Caronni et al. 2021; Subba et al. 2023); or (iii) pollen rupture in the air caused by mechanical shock from wind activity (Visez et al. 2015). In the 2022 post-season, the higher PAP values in the observed area were likely associated with osmotic shock, as the precipitation in this period was highest from all analysed years, totalling 33.2 mm.

In general, we found a significant correlation between the pollen and allergen levels which have the greatest influence on PAP. However, discrepancies between pollen and allergen concentrations were noted during some days. By investigating the backward trajectories, we identified the cases when prevailing winds could have carried bioparticles from source populations in ragweed-infested regions, such as the Pannonian Plain (Makra et al. 2005), the Rhône-Alpes region, and central areas in France (Thibaudon et al. 2014). Long-distance atmospheric transport can impact the correlation between pollen and allergen concentrations, as pollen grain rupture may lead to separate pathways for empty grains and free allergens, affecting their ratio (Moreno-Grau et al. 2016). Transport from nearby regions (such as the Pannonian Plain) tended to shift the ratio toward empty pollen grains, while longer trajectories (from the Rhône-Alpes region and central France) resulted in higher levels of free allergens. The pre-season period is particularly relevant for these analyses, as local sources of pollen and allergens are limited.

Regarding the daily variation of ragweed airborne pollen, allergen and PAP levels, we have identified a significant relationship with several meteorological variables. In general, sunny and warm weather stimulates the maturation of pollen grains in anthers, their release, and dispersion in the air (Bartková-Ščevková 2003), which explains the positive association of temperature with pollen concentration. PAP levels are also influenced by temperature, as the production of allergenic proteins in pollen is epigenetically regulated and highly temperature-dependent (Gentili et al. 2019). This relationship was negative in our findings.

The negative influence of precipitation and relative humidity on pollen and allergen concentrations, respectively, is mostly situational, since increased humidity prevents the opening of anthers and rainfall removes pollen grains from the air through the ‘wash-out’ mechanism (Olszowski 2016; Kluska et al. 2020). Even longer periods of increased moisture could be associated with a higher production of allergenic proteins in pollen grains by extending the time mature pollen remains in the anthers (Maya-Manzano et al. 2022); however, we did not observe such instances.

Wind plays a key role in the spread of pollen from anemophilous plants, causing (i) drying and rupture of anthers, (ii) lifting pollen into the air, and (iii) its dispersion (Timerman and Barrett 2021). Similar to other studies (Puc 2006; Gioulekas et al. 2004; Kasprzyk 2008; Šaulienė and Veriankaitė 2012), we found a significant positive correlation between wind speed and pollen concentration. Wind speed also facilitates the long-distance transport and resuspension of bioparticles (Grewling et al. 2016b), which can elevate pollen and allergen concentrations, potentially worsening allergy symptoms.

Allergenic molecules are produced in pollen grains during the final stage of maturation in the anthers, roughly a week before pollination (Turcich et al. 1993; Oh 2018). During this time, the influence of plant stressors, including gaseous air pollutants like CO, SO₂, and O₃ (Oksanen and Kontunen-Soppela 2021), can modify the protein content of ragweed pollen and impact its allergenic potential (Pasqualini et al. 2011; Ghiani et al. 2012; Kanter et al. 2013; Zhao et al. 2016). This influence is greatest in maturing pollen grains, which then produce a higher allergen content, as can also be observed in our results in the 5-day lag between the increased pollutant concentration and allergen and PAP levels. Since the ozone levels influence the allergen concentration with only a 1-day lag, we instead assume autocorrelation with meteorological factors influencing its formation (Stathopoulou et al. 2008).

Our observation of the association with SO₂ and CO agrees with the findings of Ghiani et al. (2012), Žiarovská et al. (2013), and Caronni et al. (2021) on *Ambrosia* and Cuinica et al. (2015) on other species. High concentrations of CO in urban areas are commonly attributed to increased traffic, which produces this pollutant through the incomplete combustion of carbon materials. Several studies have noted its association with increased cases of allergic rhinitis (Lee et al. 2003; Kim et al. 2016). The prevailing sources of SO₂ in the studied area are industrial facilities, especially petroleum refineries. Its influence on allergen production has been poorly studied so far, mostly in trees (Sousa et al. 2012; Cuinica et al. 2015). However, its potential cross-reactivity with other pollutants warrants further consideration.

## Conclusions

Our study identified some of the factors influencing the allergenicity of *Ambrosia artemisiifolia* pollen, revealing that pollen monitoring alone may be insufficient for the prophylaxis of allergic symptoms in sensitive individuals. Allergen monitoring adds valuable insights that may clarify discrepancies between pollen counts and symptom severity. We found that environmental conditions affect both annual and daily pollen and allergen levels. Pollen quantity is notably influenced by sunny, dry conditions in May, which accelerate growth, while PAP is most impacted by dry conditions in August, the main flowering period. Daily allergen concentration was statistically dependent on pollen levels, with notable deviations possibly due to long-distance pollen and allergen transport. Regression analysis indicated a significant impact of meteorological parameters on the daily data. Air pollutants, particularly CO and SO_2_, were shown to affect PAP by acting as plant stressors. The simultaneous monitoring of pollen and allergen levels and a better understanding of environmental influences could help in formulating public health strategies and contribute to the improved management of allergic conditions.

## Electronic supplementary material

Below is the link to the electronic supplementary material.

Supplementary Material 1

## Data Availability

The datasets generated during and/or analysed during the current study are available from the corresponding author on reasonable request.
