# Supplementary material for: Environmental drivers of the allergenic load caused by Ambrosia artemisiifolia pollen and its major allergen Amb a 1 in the atmosphere
Source: Int J Biometeorol. 2025 Apr 29;69(8):1885–98. doi: 10.1007/s00484-025-02932-5 (PMC12287186; doi:10.1007/s00484-025-02932-5)

**Environmental drivers of the allergenic load caused by *Ambrosia artemisiifolia* pollen and its major allergen Amb a 1 in the atmosphere**

Jana Ščevková, Matúš Žilka, Jozef Dušička, Zuzana Vašková, Jozef Kováč, Eva Zahradníková

**Fig. S1** Analysis of the residuals in the ordinary regression model for *Ambrosia* pollen concentration.


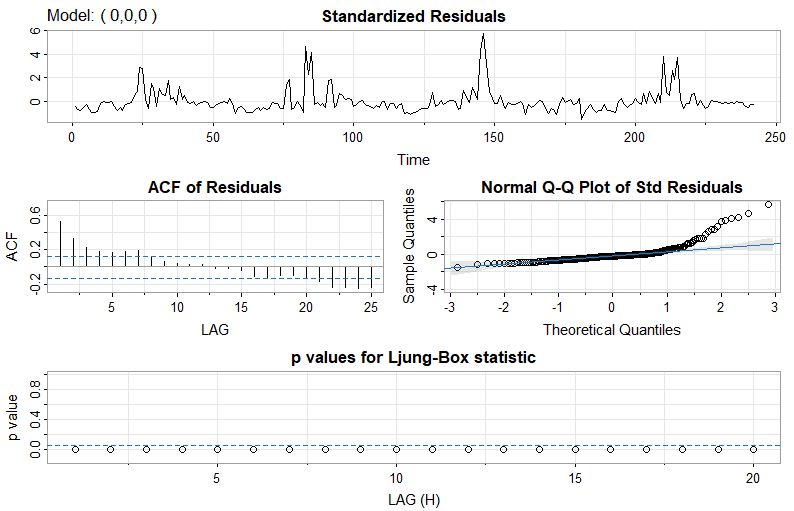


**Fig. S2** Analysis of the residuals in the ordinary regression model for the Amb a 1 allergen concentration.


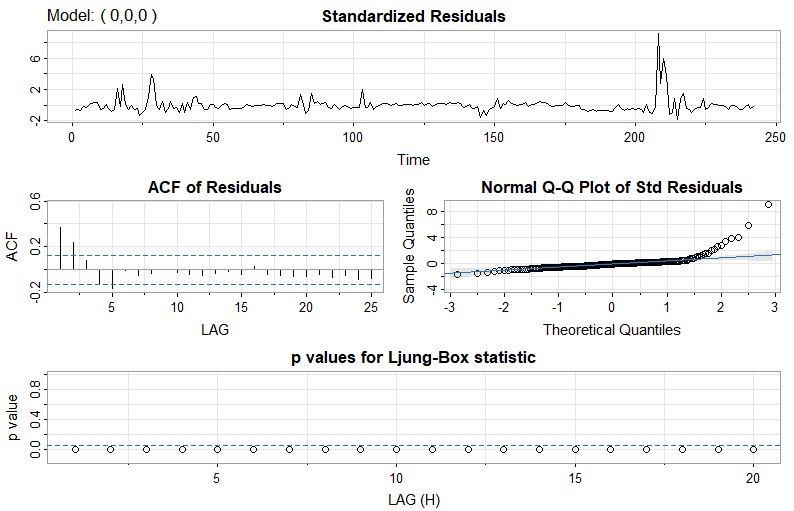


**Fig. S3** Analysis of the residuals in the ordinary regression model for pollen allergen potency (PAP).


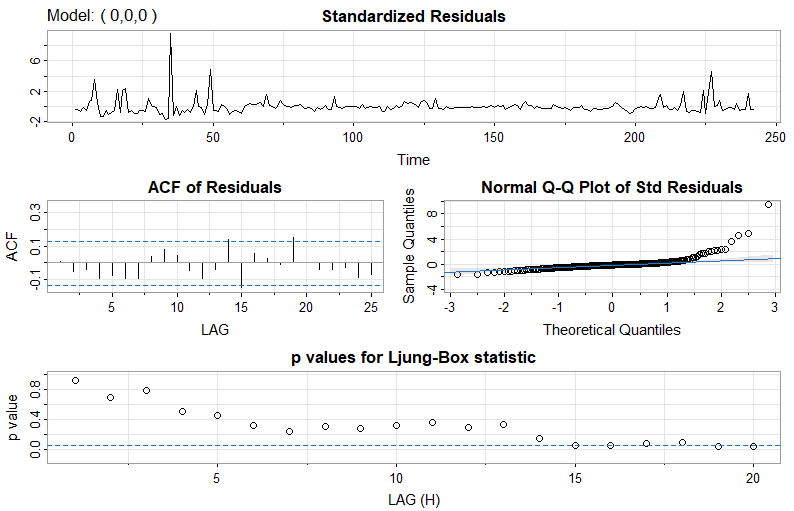


**Fig. S4** Analysis of the normalized residuals in the regression model for *Ambrosia* pollen concentration with autocorrelated errors.


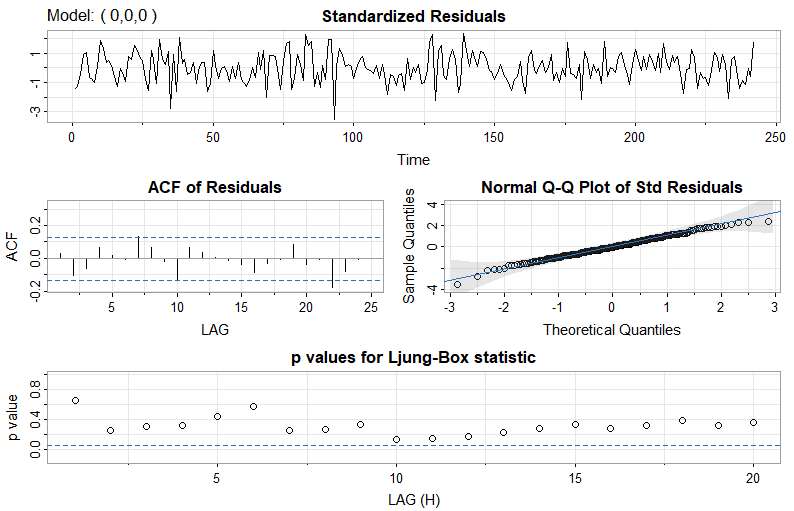


**Fig. S5** Analysis of the normalized residuals in the regression model for the Amb a 1 allergen concentration with autocorrelated errors.


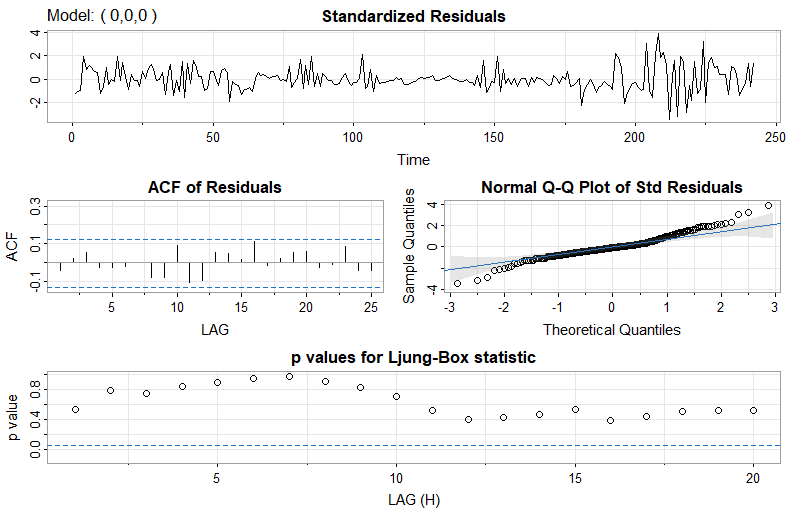


**Fig. S6** Analysis of the normalized residuals in the regression model for pollen allergen potency (PAP) with autocorrelated errors.


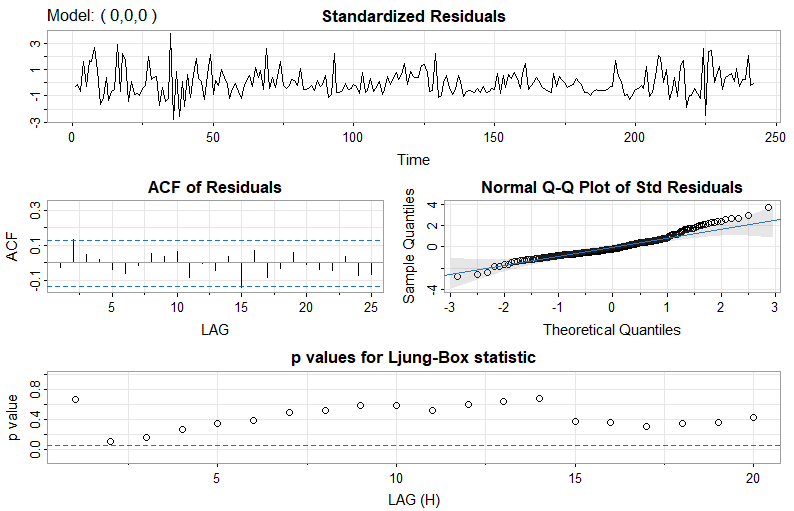

Supplement: Supplementary file 1 — Supplementary Material 1 [file 484_2025_2932_MOESM1_ESM.docx]
